# MafA Regulation in β-Cells: From Transcriptional to Post-Translational Mechanisms

**DOI:** 10.3390/biom12040535

**Published:** 2022-03-31

**Authors:** Jiani Liang, Margot Chirikjian, Utpal B. Pajvani, Alberto Bartolomé

**Affiliations:** 1 Department of Medicine, Columbia University, New York, NY 10032, USA; jliang14@student.nymc.edu (J.L.); mkc2175@cumc.columbia.edu (M.C.); up2104@cumc.columbia.edu (U.B.P.); 2 Instituto de Investigaciones Biomédicas Alberto Sols, CSIC-UAM, 28029 Madrid, Spain

**Keywords:** MafA, pancreatic beta cells, beta cell maturity, transcription factors, diabetes

## Abstract

β-cells are insulin-producing cells in the pancreas that maintain euglycemic conditions. Pancreatic β-cell maturity and function are regulated by a variety of transcription factors that enable the adequate expression of the cellular machinery involved in nutrient sensing and commensurate insulin secretion. One of the key factors in this regulation is MAF bZIP transcription factor A (MafA). MafA expression is decreased in type 2 diabetes, contributing to β-cell dysfunction and disease progression. The molecular biology underlying MafA is complex, with numerous transcriptional and post-translational regulatory nodes. Understanding these complexities may uncover potential therapeutic targets to ameliorate β-cell dysfunction. This article will summarize the role of MafA in normal β-cell function and disease, with a special focus on known transcriptional and post-translational regulators of MafA expression

## 1. Introduction

Pancreatic β-cells are critical in maintaining euglycemia by responding to blood glucose oscillations. β-cell dysfunction is a feature of diabetes mellitus which is characterized by abnormally high blood glucose levels. β-cells are responsible for the synthesis, storage and secretion of insulin, a hormone that is paramount for metabolic homeostasis. One hundred years after the successful isolation of insulin by Banting and collaborators, studies on insulin and β-cells have determined many of the factors needed for normal β-cell function, which is best defined by the appropriate expression of canonical β-cell factors involved in glucose-stimulated insulin secretion. One of these factors is the MAF bZIP transcription factor A (MafA). MafA, a large basic leucine zipper (bZIP) transcription factor (TF), derives its name from the viral oncogene *v-maf*, which was isolated from musculoaponeurotic fibrosarcoma in chicken [1]. The seven members of the MAF family were subsequently identified by homology of their bZIP domain with *v-maf* and are divided into two subgroups according to molecular size: large MAFs (Maf, MafA, MafB and Nlr) and small MAFs (MafF, MafG and MafK), which lack a transactivation domain. These factors have roles in development and terminal differentiation across multiple tissues [2,3,4], but have also been identified in disease pathogenesis [5]. For instance, MafA was first identified as the trans-acting factor binding the RIPE3b/C1 element of the insulin promoter [6,7,8] and has since been characterized as an important TF for β-cell function that is dysregulated in diabetes progression [9,10]. These studies and others emphasized the critical role of MafA for β-cell function and suggested that elucidating mechanisms of MafA regulation would deepen our understanding of β-cell pathophysiology and potentially uncover therapeutic targets for the amelioration of β-cell dysfunction and diabetes progression.

Here, we review the transcriptional and post-transcriptional regulation of MafA, with a focus on post-translational modifications (PTMs) and their effects on MafA stability and transcriptional activity. In addition, this review will discuss β-cell transcriptional targets of MafA. Other MAF factors and the role of MafA in non-β-cells are reviewed elsewhere [5]. Because of the major role MafA plays in β-cells, our goal is to summarize mouse and human studies and to provide a cohesive picture of how MafA is regulated in β-cells.

## 2. MafA Regulates β-Cell Function

The importance of MafA for glucose homeostasis was shown in MafA-deficient mice, which displayed glucose intolerance after weaning due to impaired glucose-stimulated insulin secretion (GSIS) and age-dependent diabetes progression [11,12]. MafA-deficient mice had decreased mRNA levels of *Ins1*, *Ins2*, *Pdx1*, *Neurod1* and *Slc2a2*, suggesting that MafA is essential for the transcriptional identity of β-cells. MafA works synergistically with Neurod1 and Pdx1 to transactivate the insulin promoter, which is an effect that could not be fully replicated with MafB nor Maf [13]. Along these lines, MafA promotes insulin transcription when overexpressed in rat islets [14] and when ectopically expressed in chick embryonic endoderm [15] or in other non-β-cell lines of endodermal origin [16,17].

Several studies suggest that MafA is dispensable for β-cell development but plays an essential role in β-cell maturation and glucose responsiveness of adult β-cells. In rodents, *Mafa* expression is low at birth, increasing during the postnatal period [18,19]. Interestingly, premature expression of MafA in pancreatic endocrine progenitors prevents differentiation into hormone-expressing cells [20,21], suggesting that the induction of MafA expression in β-cells is time sensitive. Consistently, in mice with whole pancreas MafA knockout (*Mafa^∆panc^*), the effects of MafA loss are not apparent until three weeks of age, when the KO mice showed lower β-cell mass, decreased insulin expression, and impaired glucose tolerance compared to controls [22]. Similarly, at twelve weeks of age in whole-body MafA knockout mice, there is a reduced β-cell:α-cell ratio, decreased islet insulin content and β-cell dedifferentiation into MafB-expressing, progenitor-like cells [12]. These results may recapitulate some aspects of normal islet development. MafB is actually the predominant large MAF protein expressed during pancreatic development in both α- and β-cells [4]. MafB can upregulate *Ins1* and *Ins2* transcription [8], but in adult mice, MafB is mainly expressed in α-cells and upregulates *Gcg* gene expression [4,23]. The decrease in β-cell MafB expression after birth is at least partly due to methylation, specifically by DNA methyltransferase 3a (Dnmt3a) which binds and represses at a region −1032 to −838 upstream of the *Mafb* transcriptional start site [24]. *Mafb^∆panc^* mice have higher blood glucose levels at P1, but two weeks after birth, blood glucose levels normalize. Likewise, at E15.5, *Mafb^∆panc^* mice have a lower number of insulin^+^ and glucagon^+^ cells compared to controls, but two weeks after birth, cell numbers became roughly the same [25]. MafB does seem to have a role in enhancing postnatal β-cell function under metabolic stress (pregnancy, high-fat diet) [24,26], but MafA remains critical for proper β-cell function and glucose homeostasis in mature β-cells, with forced MafB expression unable to compensate for MafA loss in adult mice [24].

The MAFA expression pattern in humans is similar to that observed in rodents. In human β-cells, *MAFA* mRNA expression increases postnatally in an age-dependent manner [27]. In contrast, MAFB expression in humans differs from that observed in mice. In juvenile islets (<9 years old), MAFB is expressed in a larger portion of β-cells compared to MAFA [24], and MAFB expression in human β-cells peaks at this stage, but it still can be detected though life [25]. However, similar to mice, human MAFB is required for the derivation of insulin producing β-like cells from pluripotent stem cells, suggesting a conserved role in endocrine differentiation in humans [28].

Reduced MafA expression is associated with diabetes progression in mice and human patients. In islets isolated from diabetic *db/db* mice, MafA is decreased due to hyperglycemia-associated oxidative stress and c-Jun activity [9]. Similarly, islets from subjects with type 2 diabetes (T2D) display a marked decrease in *MAFA* mRNA levels and protein expression [10,29,30]. Several studies have shown possible mechanisms for this phenomenon. In β-cells from subjects with T2D [31] and in in vitro studies mimicking oxidative stress [10], MafA was found primarily in the cytoplasm rather than properly localized in the nucleus. However, when MafA levels were “rescued” in *db/db* mice, GSIS and β-cell mass improved, suggesting that preserving MafA expression in β-cells can still mitigate diabetes progression [32]. scRNA-seq analysis of human β-cells supports this concept as well, wherein metabolically inflexible β-cells show lower MAFA activity as compared to healthy β-cells [33]. This finding is thought to align with previous patterns of T2D-induced oxidative stress, in which case MafA activity is high under healthy islet conditions but can also increase as a protective mechanism against acute oxidative stress [34]. In a different scRNA-seq analysis of human islets, β-cells co-expressing *MAFA/MAFB* had increased expression of genes related to β-cell identity, glucose metabolism and exocytosis compared to β-cells that only expressed one or neither TF [35]. Since MAFA and MAFB are capable of forming heterodimers [8,36], it is possible that the co-expression of both can enhance the β-cell maturity transcriptional program. These and other studies have documented the functional and transcriptional heterogeneity of β-cells [33,35,37]. In this regard, intra-islet MAFA expression is highly variable, which has been reported to contribute to optimal β-cell function, since homogeneous overexpression of MAFA and PDX1 through the islet led to defects in Ca^2+^ fluxes and impaired insulin secretion [38].

## 3. MafA Target Genes

MafA transcriptional activity is necessary for proper postnatal β-cell function. MafA was first identified as a TF of insulin that binds to the C1 element of the insulin gene promoter. MAF proteins, including MafA, bind to the MAF-recognition elements (MAREs), through a DNA consensus sequence TGCTGAC(G)TCAGCA [39]. However, in β-cells, the MARE sequence within the C1 element deviates slightly from the consensus sequence. The MARE sequence in C1 element of the rat and mouse *Ins2* promoter is TGCAGCTTCAGCC whereas the equivalent in the human *INS* promoter is TGCAGCCTCAGCC (the underlined nucleotides indicate deviations from the MARE consensus sequence) [7,16,40]. Interestingly, in addition to the C1 element, three other MARE sites (MARE1, MARE2 and MARE3) were also identified in the rat *Ins2* and human *INS* promoter. MafA can bind to MARE2 and MARE3 in the rat *Ins2* promoter while MafA can bind to MARE1 in the human *INS* promoter [16].

Although MafA can activate the insulin promoter alone, the addition of Neurod1 and Pdx1, which bind the E box and A box of the insulin promoter, respectively, synergistically promote insulin transcription [13]. Consistently, mice with homozygous CRISPR-Cas9-introduced mutations in the C1 box of the *Ins1* and *Ins2* gene promoters are glucose intolerant. These C1 box mutations also prevented MafA from activating the promoter in luciferase assays, further supporting the importance of MafA in upregulating the promoter of its target genes [41]. Human mutations characterized by a deletion of the C1 and E1 elements of the *INS* promoter have been shown to cause permanent neonatal diabetes, which may be attributed to the disruption of MAFA and NEUROD1 binding sites [42,43]. Other factors are also involved in this regulation. For example, Gli-similar 3 (Glis3), a Krüppel-like zinc finger transcription factor, activates insulin transcription by recruiting histone acetyltransferase Creb-binding protein (CBP) and by acting as a scaffold for the MafA, NeuroD1 and Pdx1 complex [44].

Beyond insulin, MafA increases the expression of a wide range of genes involved in β-cell function, including genes implicated in glucose sensing, insulin processing, Ca^2+^ influx and oxidative phosphorylation. Many studies have identified a plethora of MafA target genes, all having important roles in proper β-cell function, further supporting MafA’s critical role in β-cells. While some studies have found direct MafA target genes (i.e., MafA directly binds to the promoter of the target genes), other studies describe genes that are differentially expressed when MafA protein is overexpressed or silenced, without showing direct MafA binding to the target gene promoters (i.e., MafA knockout or overexpression causes a decrease or increase in the target gene expression, respectively). Some of these MafA-target genes are summarized in Table 1. The lack of promoter binding evidence does not necessarily mean MafA cannot bind to the promoter of these genes, but rather that MafA may regulate these genes through intermediaries rather than direct promoter binding.

## 4. Regulation of MafA Transcription

The first level of MafA regulation is through *Mafa* gene expression. The *Mafa* promoter consists of six regions (R1-R6), which contain highly conserved sequences 25 kb upstream the 5′ MafA untranslated region (UTR). R1 spans from −9389 to −9194, R2 spans from −8420 to −8293, R3 spans from −8118 to −7750, R4 spans from −6622 to −6441, R5 spans from −6217 to −6031 and R6 spans from −250 to +56. Of these, R3 has been shown to be most necessary, but not sufficient, for transcriptional initiation mediated by direct TF binding [56,57]. Many transcription factors bind R3 on the *Mafa* promoter, including Pax6, Nkx6.1, Nkx2.2, Pdx1, Hnf1a, Foxa2 and Isl1. Changes in these transcription factors directly correlate with *Mafa* expression. However, there are some exceptions. Neurod1, another important insulin transcription factor, can bind to R3 of the *Mafa* promoter, but *Neurod1*^−/−^ mice do not have detectable changes in MafA staining [58]. However, in a more recent study, *Neurod1*^∆endo^ mice had lower *Mafa* mRNA levels, suggesting that Neurod1 may regulate *Mafa* transcription [59]. Additionally, Pax4 is a negative regulator of *Mafa*. Pax4 overexpression limits R3-mediated promoter activity, but there is limited evidence of direct Pax4 binding to R3 via ChIP, possibly due to Pax4 regulating *Mafa* in an indirect manner [60].

Other TFs bind elsewhere in the MafA promoter or regulate *Mafa* transcription through other mechanisms, as summarized in Table 2. For instance, Onecut1, a TF involved in pancreatic endocrine development that is upregulated in adult *db/db* mice, acts as a negative regulator of *Mafa* transcription by preventing Foxa2 from interacting with the *Mafa* promoter, highlighting the importance of the transcriptional regulation of *Mafa* in metabolic disease states [61].

Foxo1, a TF involved in a number of metabolic pathways, is inactive in resting β-cells, but after acute high glucose or H_2_O_2_ exposure, translocates to the nucleus where it enhances *Mafa* expression by binding the forkhead element in the *Mafa* promoter [34,66]. Overexpression of Foxo1 in mouse β-cells increased *Mafa* mRNA and decreased age-dependent β-cell mass decline [67]. Conversely, the loss of Foxo1 is associated with reduced expression of MafA and other important β-cell TFs, eventually leading to a loss of cell identity in conditions of stress [68]. Other mechanisms are also at play. For example, active thyroid hormone (T_3_), bound to the thyroid hormone receptor, directly transactivates the *Mafa* promoter [64]. Similarly, transcriptional elements downstream of GLP1R, such as CREB-transcription factors and CREB-regulated transcription coactivator 2 (Crtc2), also regulate *Mafa* transcription. CREB constitutively binds the cAMP response element of the *Mafa* promoter, while Crtc2 binds the *Mafa* promoter following forskolin treatment, enhancing *Mafa* transcription. This MafA expression-potentiating mechanism is impaired by chronic high glucose levels [65].

The transcription of *Mafa* in β-cells is also sensitive to acute changes in glucose levels. In low glucose conditions, *Mafa* mRNA levels are low, but after acute high glucose exposure, *Mafa* transcription increases. This glucose-dependent transcriptional regulation is mediated by the hexosamine biosynthesis pathway and is thought to be independent of glycolysis [69]. However, under metabolically stressful conditions (i.e., hyperglycemia), *Mafa* transcription paradoxically decreases [10,70]. Oxidative stress causes lower binding of certain TFs (Pdx1 and Nkx6.1) to the *MafA* promoter, which at least partly contributes to lower *Mafa* mRNA [10]. Palmitate, a fatty acid that inhibits insulin gene expression, also decreased glucose-stimulated *Mafa* transcription, possibly by decreasing Pdx1 nuclear localization and binding to the *Mafa* promoter [71]. Additionally, palmitate may increase c-Jun N-terminal kinase (JNK) activity, which previously was found to correlate negatively with *Mafa* transcription under low glucose conditions, thereby causing a decrease in *Mafa* transcription [72,73]. Likewise, aldosterone, a key player in the renin–angiotensin–aldosterone system (RAAS), can mediate β-cell dysfunction by enhancing JNK activity, causing a decrease in *Mafa* at the transcriptional level [74].

*Mafa* gene expression is also affected by non-coding RNAs (ncRNA). A number of studies have identified microRNAs (miRNA)—short ncRNAs that base-pair with their target mRNA at the 3′-untranslated region (UTR) to promote mRNA instability and repression in protein translation [75]—that regulate *Mafa* gene expression [76,77,78,79]. One study found that thioredoxin-interacting protein (Txnip) decreases insulin gene expression by upregulating miR-204, which targets the 3′-UTR of *Mafa* mRNA, resulting in mRNA degradation and decreased protein translation [76]. However, this effect could not be replicated in human islets [80], likely due to a mismatch between miR-204 and its target 3′-UTR of human *MAFA* that is not present in the 3′-UTR of rat *Mafa* [80]. Other miRNAs that affect *Mafa* gene expression include miR-149, which negatively regulates *Mafa* after arsenite exposure [77]; miR-30d, which positively regulates *Mafa* by inhibiting Mitogen-activated protein 4 kinase 4 (Map4k4) [78]; and miR-24, which negatively regulates *Mafa* [79]. Most of these miRNAs have been studied in mouse islets or cell lines; despite sequence homology [78], effects in human β-cells still require experimental validation.

*Mafa* is also affected by long non-coding RNAs (lncRNA). LncRNAs are transcripts of >200 bp that that lack protein-coding potential, but have biological roles through chromatin remodeling, imprinting induction, regulation of splicing and mRNA translation [81]. For example, the lncRNA *Meg3* binds Ezh2, a methyltransferase, which causes suppression of gene expression of three repressive transcriptional regulators (Rad21, Smc3, and Sin3a) via promoter methylation, culminating in an increase in *Mafa* expression [82]. In humans, the *MEG3* promoter is hypermethylated in islets from human subjects with T2D, suggesting that *MEG3* expression is downregulated in T2D, which may contribute to decreased *MAFA* expression [83,84].

## 5. MafA Post-Translational Modifications

**Figure 1:**
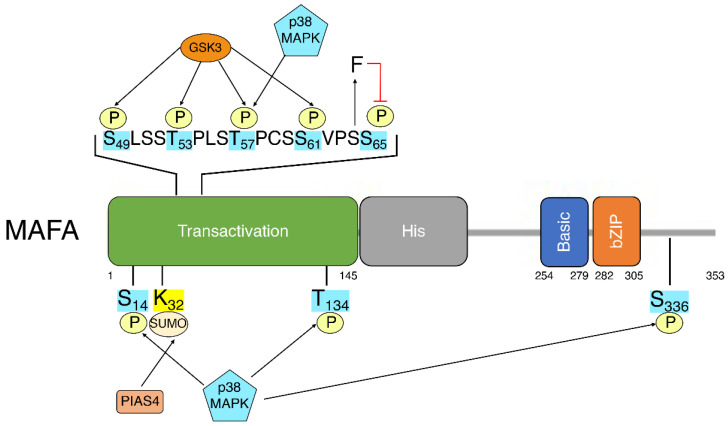
Linear schematic of MAFA depicting MAFA domains and known post-translationally modified residues. Transactivation: transactivation domain. His: histamine-rich region. Basic: basic domain. bZIP: Leucine zipper.

The previously discussed studies indicate the importance and potential mechanisms of *Mafa* transcriptional regulation. Post-translational modifications (PTMs) add a second layer of regulation, allowing for dynamic changes in MafA activity or stability in response to changes in β-cell environment. While most documented PTMs are found in the N-terminal transactivation domain of MafA [85], other regions of MafA, such as the extended homology region, the basic domain and the leucine-zipper, affect DNA recognition and binding and are also likely affected by the addition of PTMs [5] (Figure 1).

### 5.1. Phosphorylation

The most studied PTM of MafA is phosphorylation. In fact, even before MafA was identified as the TF binding to the C1 box of the insulin promoter, phosphorylation was deemed necessary for DNA binding [86]. MafA is highly phosphorylated under basal conditions, which is reduced by H_2_O_2_-induced oxidative stress and likely other stress. Phosphorylation of MafA increases MafA transcriptional activity and enhances its interactions with binding partners such as Neurod1 [87], which allows for MafA dimerization [85], and increases DNA binding [88]. A number of MafA phosphorylation sites have been identified through mass spectrometry [85], and subsequently characterized [89]. Of these, phosphorylation of Ser65, discussed more extensively later in this review, has been the most studied as it increased MafA transactivation but paradoxically decreased protein stability [90]. However, numerous other Ser/Thr residues are also phosphorylated, including Thr134 by p38MAPK [91], which is increased with oxidative stress and leads to MafA degradation [92,93]. Interestingly, in addition to decreasing *Mafa* mRNA levels, aldosterone can also mediate β-cell dysfunction by enhancing p38MAPK-induced MafA phosphorylation, in turn causing MafA degradation [74].

The most studied phosphorylated residues are those in the MafA transactivation domain associated with glycogen synthase kinase 3 (GSK3) [94,95]. Most GSK3 target proteins contain a “priming” residue that must be phosphorylated by another kinase before GSK3 can phosphorylate its target residues. GSK3 often phosphorylates its targets in a sequential manner, on every fourth residue that is serine or threonine after the priming residue is phosphorylated [96]. The mechanism for GSK3-mediated MafA phosphorylation is no different. GSK3 cannot phosphorylate its target residues until Ser65 of MafA is phosphorylated by an unknown “priming” kinase. After Ser65 is phosphorylated, GSK3 sequentially phosphorylate MafA on Ser61, Thr57, Thr53 and Ser49. GSK3-mediated phosphorylation not only increases MafA activity but also increases MafA degradation. The S65A mutation, which prevents the unknown priming kinase from phosphorylating MafA, or mutating all the residues targeted by GSK3, results in a very stable MafA protein as compared to WT MafA [95]. Consistent with the importance of this regulation, a recent study showed that MafA stability is also affected by the GSK3-regulator Fermt2 (also known as Kindlin-2), an integrin activator [97]. Fermt2 increases Gsk3b Ser9 phosphorylation, which decreases Gsk3b activity. Thus, β-cell-specific Fermt2-knockout mice show reduced islet MafA and impaired GSIS, leading to glucose intolerance. The study did not investigate whether Fermt2 decreased Gsk3b-mediated MafA phosphorylation, but the results suggest that MafA regulation by Fermt2 is important in the intricate regulation underlying MafA stability and activity.

Interestingly, another study identified MAFA S64F missense mutations in two families, which led to hypophosphorylation of MafA [98]. Phenotypically, males with this mutation were more likely to develop T2D, while the females were more likely to develop hyperinsulinemia-induced hypoglycemia. These phenotypes were replicated in mice as well [99]. Molecularly, S64F mutation increased MafA stability under both low and high glucose conditions and decreased phosphorylation, suggesting the hypothesis that S64F may prevent the priming kinase from phosphorylating Ser65, possibly through steric inhibition. More studies would be needed to further support the hypothesis, but this work highlights the importance of MafA phosphorylation in maintaining proper β-cell function in humans.

### 5.2. SUMOylation

Small ubiquitin-like modifier (SUMO) has been shown on MafA lysine residues. Despite its name, SUMOylation does not target proteins for proteasomal degradation but rather affects protein function, stability and transport. SUMOylation occurs on lysine residues at a consensus Ψ-K-X-E motif, where Ψ represents a hydrophobic amino acid. SUMOylation of Lys32 occurs at this motif (VK_32_KE) [100,101], and similarly in MafB and Maf at Lys32 and Lys33, respectively [100]. In immortalized β-cell lines (MIN6 and INS-1), low-glucose conditions and oxidative stress caused increased endogenous MafA SUMOylation, suggesting a physiological role of this PTM [101]. SUMOylation of MafA resulted in a decrease in MafA transcriptional activity. Conversely, preventing MafA SUMOylation with a K32R mutation resulted in increased transactivation of the insulin promoter [100,101]. Intriguingly, MafA SUMOylation did not significantly impact protein stability [100]. These results suggest that in β-cells, SUMOylation may act as a mechanism to decrease MafA-mediated transactivation, but not stability, in low-glucose and oxidative conditions. Consistently, the E3 SUMO protein ligase protein inhibitor of activated STAT4 (PIAS4) enhanced MafA SUMOylation and negatively regulated the insulin promoter, though PIAS4 interaction with the bZIP domain of MafA is sufficient for transcriptional repression, independently from SUMOylation [102].

### 5.3. Ubiquitination

Ubiquitin acts in pleiotropic ways, depending on the pattern of ubiquitination. Polyubiquitination usually marks a protein for proteasomal degradation, but monoubiquitination can regulate DNA repair and gene expression [103,104]. MafA has been shown to be polyubiquitinated in the lysine-rich C-terminal domain of MafA, causing MafA proteasomal degradation [90]. Currently, no ubiquitinated lysine on MafA has been identified thus far, and no study, to our knowledge, has investigated the role MafA monoubiquitination.

MafA ubiquitination is largely contingent on MafA phosphorylation. A number of studies have shown that blocking MafA phosphorylation, in particular at S65, significantly decreases ubiquitination and increases MafA half-life [90,94,95]. Commensurately, GSK3 inhibitors also increase MafA half-life [94,95]. Furthermore, proteasomal activation with PA28γ induces degradation of WT MafA but does not induce degradation of non-phosphorylatable MafA mutant nor dephosphorylated MafA due to GSK3 inhibition, further suggesting that phosphorylation is a necessary step to MafA proteasome-dependent degradation [105].

Although MafA ubiquitin status is dependent on its phosphorylation status, there are mechanisms that protect MafA phosphorylation from degradation. K(lysine) acetyltransferase 2B (Kat2b), also known as p300/CBP-associated factor (PCAF), is believed to enhance the transactivation potential of GSK3-phosphorylated MafA by protecting it from degradation [94]. Kat2b decreases MafA ubiquitination, thereby increasing the transcriptional activity of MafA through stabilization. In the context of metabolic disease, disruption of MafA–Kat2b interaction results in the loss of β-cell maturity. Our studies investigating Notch signaling in β-cells found that forced Notch signaling in β-cells caused MafA degradation, leading to impaired GSIS and thereby glucose intolerance. We found that active Notch expression reduces Kat2b–MafA complex and that Kat2b silencing alone is able to cause a loss of MafA and impaired GSIS [106]. These findings emphasize the importance of Kat2b in MafA stability through preventing ubiquitination.

**Figure 2:**
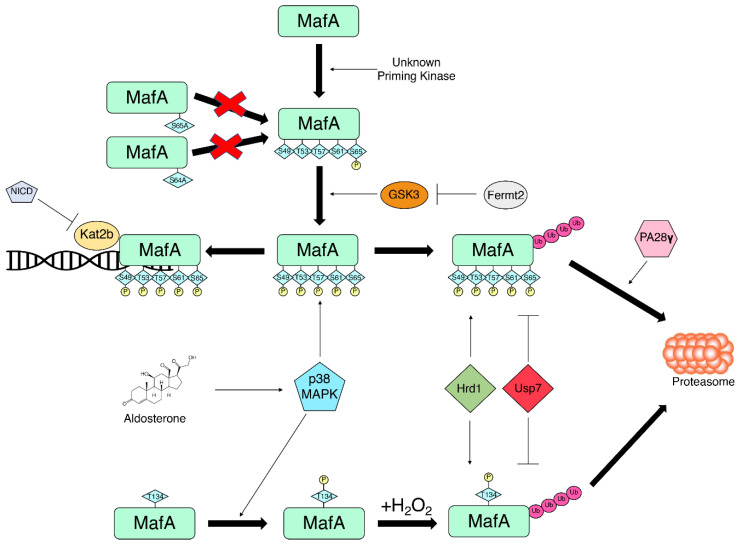
Regulators of MafA phosphorylation and ubiquitination fine-tune the balance between MafA transcriptional activity and MafA degradation.

However, MafA ubiquitination may be even more complex, as two novel regulator proteins for MafA have also been identified: ubiquitin-specific protease 7 (USP7) and HMG-CoA reductase degradation protein 1 (HRD1), also known as synoviolin. USP7 is a ubiquitin protease that cleaves ubiquitin off its substrate while HRD1 is an endoplasmic reticulum-resident E3 ubiquitin ligase [107,108]. Re-CLIP/MS analysis of endogenous MafA-binding partners in a β-cell line (βTC-3 cells) identified Usp7 [109]. Co-expression of MafA with USP7 decreased MafA ubiquitination and stabilized MafA [107]. By contrast, HRD1 increased the ubiquitin status of MafA while HRD1 knockdown decreased MafA ubiquitination and increased MafA stability [108]. The identification of USP7 and HRD1 as MafA ubiquitin protease and E3 ligase, respectively, provides more novel therapeutic targets that could potentially stabilize MafA to increase MafA target gene expression (summarized in Figure 2).

### 5.4. Other PTMs

MafA may be regulated by other PTMs. MafG can be acetylated at Lys53, Lys60, Lys71 and Lys76, which increases its transcriptional activity [110]. Indeed, as mentioned before, Kat2b, an acetyltransferase, regulates MafA, but specific deacetylases that regulate MafA have not yet been identified. In addition, MafA acetylation has not yet been described. Similarly, although UDP-N-acetylglucosaminyl transferase (OGT), which causes O-glycosylation on its targets, is involved in glucose-dependent *Mafa* gene expression [69], no evidence has yet been shown for O-glycosylation of MafA [95]. Studies investigating MafA PTMs deepened our understanding of MafA regulation. Future studies on novel MafA PTMs or novel findings on known MafA PTMs can uncover potential therapeutic targets to ameliorate β-cell dysfunction.

## 6. Conclusions

In the last two decades, many studies have investigated MafA in the context of β-cell function. Since its discovery as a regulator of insulin transcription, MafA has also been identified as a TF for numerous other genes involved in the acquisition of β-cell functional maturity status, including genes involved in glucose sensing and insulin secretion. Consistently, the loss of β-cell MafA in mice leads to glucose intolerance and diabetes progression. In humans, decreased β-cell MAFA correlates with T2D, further supporting the importance of MAFA in maintaining β-cell function. Many studies have identified regulators of *Mafa* transcription, but MafA also shows important post-translational regulation through phosphorylation, SUMOylation and ubiquitination. Further elucidation of the molecular underpinnings of MafA regulation may provide novel therapeutic targets for the treatment and prevention of diabetes.

## Figures and Tables

**Table 1:** MafA target genes. (*) denotes evidence that MafA directly binds target gene promoter.

Gene Symbol	Gene Name	Gene Function	Reference
Cacng4 *	Voltage-dependent calcium channel gamma-4 subunit	Enhances L-type Ca^2+^-mediated Ca^2+^ entry into β-cell	[45]
Chrnb4 *	Cholinergic receptor nicotinic beta 4	Subunit of the nicotinic acetylcholine receptor	[46]
Cox6a2 *	Cytochrome C oxidase subunit 6A2	One out of 13 subunits of cytochrome C oxidase complex (Complex IV), the last enzyme in the electron transport chain	[47]
G6pc2 *	Glucose-6-phosphate catalytic subunit related protein	Islet-specific enzyme that hydrolyzes glucose-6-phosphate, limits basal insulin secretion	[48]
*Gck*	Glucokinase	Phosphorylates glucose to glucose-6-phosphate in pancreatic islets and hepatocytes. Considered the β-cell glucose sensor	[49]
*Glp1r*	Glucagon-like peptide 1 receptor	Receptor for Glucagon-like peptide 1 (Glp1), a stimulator of insulin secretion	[49]
Ins1 *	Insulin I	One of two insulin genes in mouse, on chromosome 19	[11]
Ins2 *	Insulin II	One of two insulin genes in mouse, on chromosome 7	[11]
Maob *	Monoamine oxidase B	Metabolizes monoamine neurotransmitters, specifically benzylamine, dopamine and phenylethylamine	[50]
*Neurod1*	Neurogenic differentiation 1	Transactivator of genes important for β-cell maturation and function, including insulin	[49]
*Nkx6.1*	NK6 homeobox1	TF involved in β-cell development and regulation of genes involved in mature β-cell function	[49,51]
*Pcsk1*	Proprotein convertase subtilisin/kexin type 1	Proprotein convertase, which processes proinsulin in β-cells	[49]
*Pcx*	Pyruvate carboxylase	Catalyzes the conversion of pyruvate to oxaloacetate	[49]
Pdx1 *	Pancreatic and duodenal homeobox 1	TF important pancreas development and for mature β-cell function	[49,52]
*PPP1R1A*	Protein phosphatase 1, regulatory inhibitor subunit 1A	Regulates cAMP/PKA signaling pathway Promotes Glp1-induced GSIS	[53]
*Prlr*	Prolactin Receptor	Involved in increasing β-cell mass during pregnancy	[54]
Slc2a2 *	Solute Carrier Family 2 Member 2	Glucose transporter 2, transmembrane glucose transporter with a high *K_m_* for glucose	[49,55]
*Slc80a8*	Solute carrier family 30 member 8	Zinc transporter on insulin granules in β-cells	[19,46]

**Table 2:** Known transcriptional regulators of *Mafa* gene expression.

Protein Symbol	Protein Name	*Mafa* Transcription Mechanism	Reference
Pax6	Paired box protein Pax-6	Binds R1, R3 and R6 of the *Mafa* promoter	[58]
Nkx6.1	NK6 homeobox 1	Binds R3 of the *Mafa* promoter	[58]
Nkx2.2	NK2 homeobox 2	Binds R3 of the *Mafa* promoter	[57]
Pdx1	Pancreatic and duodenal homeobox 1	Binds R3 and R6 of the *Mafa* promoter	[57,58]
Hnf1a	Hepatocyte nuclear factor 1-alpha	Binds R3 of the *Mafa* promoter	[56]
Foxa2	Forkhead box A2	Binds R3 of the *Mafa* promoter	[57]
Isl1	Insulin gene enhancer protein ISL-1	Binds R3 of the *Mafa* promoter	[62]
Neurod1	Neurogenic differentiation 1	Binds R3 of the *Mafa* promoter	[57]
Pax4	Paired box protein Pax-4	Negative regulator of *Mafa*, potentially by interfering other factors from binding R3 of the *Mafa* promoter	[60]
Mafb	Transcription factor MafB	Binds R3 of the *Mafa* promoter	[58,63]
Onecut1	One cut domain, family member 1	Prevents Foxa2 from binding to the *Mafa* promoter	[61]
Foxo1	Forkhead box O1	Binds to the forkhead element of the *Mafa* promoter	[34]
Thra	Thyroid hormone receptor alpha	Binds to Thyroid hormone response element (TRE), which are located from −1927 to −1946 and from +647 to +659 (named Site 2 and Site 3, respectively)	[64]
CREB	cAMP responsive element binding protein	Constitutively binds to the cAMP response element (CRE), spanning from −1342 to −1346, of the *Mafa* promoter	[65]

## Data Availability

Not applicable.
